# Acknowledgment to the Reviewers of *Vision* in 2022

**DOI:** 10.3390/vision7010007

**Published:** 2023-01-19

**Authors:** Vision Editorial Office

**Affiliations:** MDPI AG, St. Alban-Anlage 66, 4052 Basel, Switzerland

High-quality academic publishing is built on rigorous peer review. *Vision* was able to uphold its high standards for published papers due to the outstanding efforts of our reviewers. Thanks to the efforts of our reviewers in 2022, the median time to first decision was 30.5 days and the median time to publication was 65 days. Regardless of whether the articles they examined were ultimately published, the editors would like to express their appreciation and thank the following reviewers for the time and dedication that they have shown *Vision*:
Ahmed AbdelghanyJuliette E. McGregorAkira ObanaKang-Ming ChangAlessandro MeduriKatarzyna NowomiejskaAlessio FacchinKazuo IchikawaAlfonso Vasquez-PerezKevin PatersonAlice Verticchio VercellinKjetil Falkenberg HansenAlicia Ruiz-PomedaKristopher R. GenschmerAlina GheorgheKrzysztof SchabowiczAmedeo D’AngiulliKwok Ping ChanAmir KheradmandLaurence HarrisAna Tajadura-JimenezLei WanAndré MermoudLeo FleishmanAndreas EichlerLuca BattagliniAndrzej GrzybowskiLuigi TameÁngel De-Juanas OlivaLuke ChongAnita Lyssek-BorónMael LeverAnna PiroManfred MarschallAnna Przekoracka-KrawczykManuel PereaArjen AlinkMarco BertaminiArne Lykke LarsenMare KoitArne OhlendorfMarek HamplArthur ZhaoMarialuisa MartelliBaingio PinnaMarina GregoricBenjamin KnierMario DalmasoBhagyashri BurguteMark J. SimcoeBiswajit PadhyMark W. GreenleeBrad BarnettMartin HowBruno RichardMarty BanksCalvin EiberMassimo BusinCarlos Ferrás SextoMaurizio CammalleriCecilia PerinMaxim BakaevChong-Bin TsaiMd Manjurul AhsanChris OrietMelinda Y. ChangChristoph FaschingerMengxi ShenChristoph M. FriedrichMichael D. CrosslandClara Martinez-PerezMichael HoffmannClaudio BucoloMichael J. LipsonClémence BonnetMichał S. NowakCord HuchzermeyerMichele LanzaDanial RoshandelMiguel TeusDaniel Josef LindeggerMile BošnjakDaniel López MaloMinglang YinDavid Manzano SánchezMohammad Shokrolah ShiraziDmitrii S. MaltsevMohsen Mosayebi-SamaniDoreen SchmidlMuneeswar NittalaDoug D. ChungNehal ElsherbinyDušica PahorNicola Di StefanoEleftherios AnastasopoulosNicolae GicaElena DinteNicoleta AntonEleonora MauriziNikos KonstantinouEliya LevingerNingli WangEmily HandNorman M. SpivakEvan PalmerOlaia Lucas-JiménezEwald LindnerPadmanabhan PattabiramanFrancesco LenaPastore MassimilianoFrancisco AlonsoPatrick CavanaghFrank H. DurginPaul HibbardGaganpreet KaurPaul LintonGeorges DellatolasPavol BožekGeorgios LabirisPeter KoulenGerd GigerenzerPushpa Raj JoshiGerd U. AuffarthQi FangGiacomo CollettiRafael IribarrenGiancarlo MontaniRalph WeidnerGiovanni William OliverioRan DuGiuseppe CirilloRebecca FullerGrainne ScanlonRicardo Bernardez-VilaboaGregory DeAngelisRicardo F. FraustoGrzegorz ŁabuzRobert J. EnnisGuido MaielloRobert L. WhitwellGysbert van SettenRobert S. AllisonHafiz Tayyab RaufRoberto BellucciHakan KaymakRohan Bir SinghHaluk OgmenRosa BocciaHamdy AbdelkaderRuobing QianHarold BedellRupesh Kumar ChikaraHaruka AbeSamantha R. De SilvaHe-Sheng WangSerenella D’IngeoHirokazu SakaguchiSergio MorraHortensia T. Sanchez-TocinoShao-Bin WangHuei-Jane LeeSinwan CheungHun-Ju YuSiyi ChenIoannis HalkiadakisSreelakshmi VasudevanIrene SperandioTae Keun YooIvan MatveevTatu RojalinIvana MravicicTejas KarhadkarJames J. WolffsohnThomas A. BuseyJamie M. BogleThomas SchmidtJared CoburnTimothy J. VickeryJavier MartínTina McKayJee Taek KimTomislav KuzmanJeffrey BaraTsung-Jen WangJeffrey J. HuangVasile-Gabriel IanaJerónimo González-BernalVida DemarinJin BaiWaldemar SwiderskiJinjun XiaWladimir KirschJoanna KonopińskaYokrat TonJovana BjekićYuntao HuJukka HyonaZhong-Lin Lu


